# *Eurycoma longifolia* (Jack) Improves Serum Total Testosterone in Men: A Systematic Review and Meta-Analysis of Clinical Trials

**DOI:** 10.3390/medicina58081047

**Published:** 2022-08-04

**Authors:** Kristian Leisegang, Renata Finelli, Suresh C. Sikka, Manesh Kumar Panner Selvam

**Affiliations:** 1 School of Natural Medicine, Faculty of Community and Health Sciences, Bellville, Cape Town 7535, South Africa; kleisegang@uwc.ac.za; 2 CREATE Fertility, 150 Cheapside, London EC2V 6ET, UK; finelli.renata@gmail.com; 3 Department of Urology, Tulane University School of Medicine, New Orleans, LA 70112, USA; ssikka@tulane.edu

**Keywords:** *Eurycoma longifolia*, Tongkat ali, testosterone, male, hypogonadism

## Abstract

*Background and Objectives*: Male hypogonadism is a clinical disorder characterized by reduced serum testosterone in men. Although treatment using herbal medicines, including *Eurycoma longifolia,* has been investigated, the benefits remain unclear. This study aims to investigate the efficacy of *E. longifolia* as a sole intervention to increase testosterone levels in males. *Materials and Methods*: We conducted a systematic review and meta-analysis of randomized clinical trials (RCTs) according to the PRISMA guidelines. Relevant articles were retrieved from the databases PubMed, Scopus, Web of Science, Cochrane, Ovid/Embase, and Google Scholar. *Results:* After literature screening, a total of nine studies was included in the systematic review. Five RCTs were included in the meta-analysis. A significant improvement in total testosterone levels after *E. longifolia* treatment was mostly reported in both healthy volunteers and hypogonadal men. The random model effect revealed a significant increase (SMD = 1.352, 95% CI 0.565 to 2.138, *p* = 0.001) in the total testosterone levels in men receiving *E. longifolia* supplementation, which was confirmed in the hypogonadism subgroup. *Conclusions*: This systematic review and meta-analysis of the literature supports the possible use of *E. longifolia* supplementation for enhancing testosterone production. Although more research is required before its use in clinical practice, this may represent a safe and promising therapeutic option, particularly in hypogonadal men.

## 1. Introduction

Male hypogonadism is a clinical disorder that arises from a failure of the testes to produce adequate levels of testosterone, mainly mediated through a disruption of the hypothalamic–pituitary–gonadal (HPG) axis [1]. Hypogonadism is estimated to affect 1.2–12.8% of middle-aged and older men in the general population, with increasing incidence and burden on healthcare services [2]. The biochemical definition of hypogonadism remains unclear; however, a 300 ng/dL threshold has been generally recommended as the lowest limit of normal [2], with general consensus that testosterone levels above 350 ng/dL do not require treatment. Conversely, patients with testosterone levels less than 230 ng/dL usually benefit from testosterone replacement therapy (TRT) [3,4]. Hypogonadism is further classified based on gonadotropin levels as primary (hypergonadotropic) or secondary (hypogonadotropic) [2].

Common clinical features of hypogonadism include reduced libido and erectile dysfunction, male factor infertility, obesity with reduced lean body mass, reduced bone density, fatigue, and depression [2]. Lower levels of testosterone in healthy men is a predictor of co-morbidities [5,6], such as obesity, metabolic syndrome, type 2 diabetes mellitus, cardiovascular disease, and osteoporosis. Clinically, management of hypogonadism is focused on TRT that can improve sexual dysfunction and well-being, reduce obesity, increase lean body mass, and increase bone density [7]. Although TRT has become a multimillion dollar market, only about 10% of men with hypogonadism in the USA and Europe are being treated with TRT [8]. Furthermore, TRT is contraindicated in patients who want to preserve fertility and those with prostate carcinoma, benign prostatic hyperplasia and/or lower urinary tract symptoms, high serum prostatic specific antigen (PSA) levels, obstructive sleep apnoea, or patients with a history of myocardial or cerebrovascular stroke [1,2]. The benefits compared to risks of long-term TRT remain unclear in men treated for mild hypogonadism or age-related hypogonadism [7]. Hence, many physicians perceive the risks of TRT to be high.

Numerous herbal medicines have shown potential to increase serum testosterone levels and benefit sexual function and fertility [6,9,10]. In particular, *Eurycoma longifolia* Jack (Tongkat ali or Malaysian ginseng) has been traditionally used for management of male sexual dysfunction and infertility [11,12,13,14]. In addition, *E. longifolia* also possesses other medical properties, such as positively impacting athletic performance and muscular bulk, reducing adiposity, stimulating appetite, and treating fatigue, malaria, diabetes, anxiety, osteoporosis, cancer, constipation, and peptic ulcers [11,12,13,14,15].

*E. longifolia* is reported to improve libido and is being used as a common ingredient in more than 700 herbal or nutraceutical products marketed as aphrodisiacs [16,17,18,19]. Recent systematic reviews have summarized the benefits of *E. longifolia* to men’s reproductive health [20,21]. However, it remains unclear whether *E. longifolia* improves serum testosterone levels in men. With a high burden of hypogonadism in the general population, *E. longifolia* may provide benefit in increasing serum testosterone in men. Therefore, this study aims to investigate the efficacy of *E. longifolia* to increase testosterone in males, using a meta-analysis of available randomized clinical trials (RCTs).

## 2. Materials and Methods

### 2.1. Search Strategy and Risk-of-Bias Assessment

A systematic review and meta-analysis was conducted according to the Preferred Reporting Items for Systematic Reviews and Meta-Analyses (PRISMA) guidelines [22]. The following keyword combinations and Boolean operators were used: (“Tongkat” OR “*Eurycoma*” *OR* “*longifolia*” *OR* “*pasak bumi*”) AND (“testosterone”). The literature search was performed on 10 July 2021 to search articles published until that date on the databases PubMed, Scopus, Web of Science, Cochrane, Ovid/Embase, and Google Scholar. These databases were searched to identify clinical trials investigating the use of *E. longifolia* extraction as a sole intervention in adult males and reporting pre- and post-treatment serum testosterone as an outcome.

Animal studies, in vitro and in silico studies, meta-analyses, reviews, case reports, letters, editorials, comments, and non-English-language publications were excluded. In addition, the duplicate articles retrieved from different databases were removed. The remaining articles were screened independently for titles and abstracts by two authors (R.F. and K.L.) to exclude non-relevant studies, while any disagreement was settled by an additional researcher (M.K.P.S.). Irrelevant articles were removed, and full-text articles were screened for eligibility based on the inclusion and exclusion criteria. Data were extracted by using a precompiled Excel file, and included the study setting, cohort description, details of experimental herbal intervention extraction, dosage and duration of the experimental intervention, total (ng/dL) and free (ng/dL) serum testosterone measurements for cases and controls before and after treatment, along with sex hormone binding globulin (SHBG) (nmol/L) and dehydroepiandrosterone (µg/mL) assessments as secondary outcomes. In case numerical variables were not reported in the manuscripts, respective study authors were contacted via email and requested to share their results for inclusion in the meta-analysis. For the randomized trials included in this meta-analysis, the quality and the risk of bias were assessed independently by two authors (R.F., K.L.) using version 2 of the Cochrane risk-of-bias tool for randomized trials [23]. Both observational and randomized studies were included in the systematic review, whereas the meta-analysis was based on RCTs only.

### 2.2. Statistical Analysis

Meta-analysis was performed by using mean and standard deviation for continuous outcome variables (testosterone levels). Based on significance of Cochran’s Q value and I^2^ (inconsistency) statistics, either fixed or random effects models were used to analyze the pooled data. A subgroup analysis was conducted according to the testosterone levels of study subjects. Studies with subjects having low testosterone levels (<300 ng/dL) prior to treatment with *E. longifolia* were considered as showing hypogonadism. Furthermore, publication bias among the studies was assessed by Egger’s test [24] and Begg’s rank test [25]. All analyses were performed by using MedCalc Software (version 20.019, Ostend, Belgium).

## 3. Results

**Figure 1:**
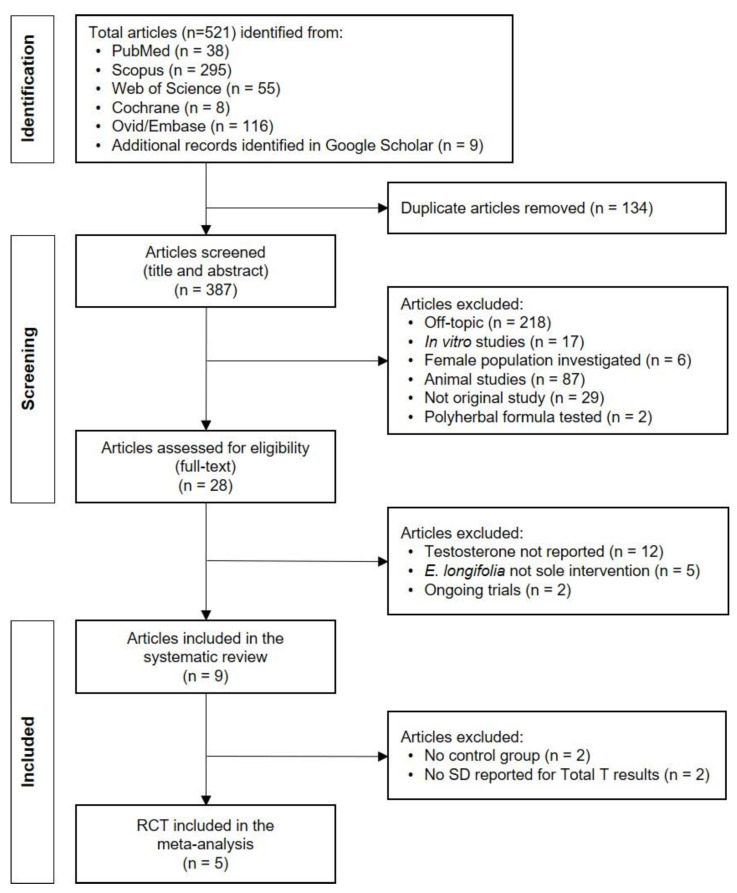
Flow diagram reporting the search strategy. SD: standard deviation.

The search strategy identified a total of 521 articles (Figure 1). After removing duplicates (*n* = 134), the titles and abstracts of 387 articles were screened for inclusion, with a further 359 studies being excluded. The full text of identified articles (*n* = 28) was assessed based on the inclusion criteria. A total of nine studies was included in this systematic review, while five RCTs were included for meta-analysis.

### 3.1. Systematic Review

The literature screening identified a total of nine studies published between 2012 and 2021 that investigated the effect of *E. longifolia* on serum testosterone levels in men. Of these, two were comparative (pre- vs. post-) prospective studies, and five were double-blind, controlled trials (of these, four were randomized). Two additional RCTs were published as doctor of philosophy degree (PhD) dissertations. Characteristics of the included studies (*n* = 9) are reported in Table 1. There were three studies that investigated a male population affected by hypogonadism [26,27,28], while six studies included a population of healthy men with normal testosterone levels [29,30,31,32,33,34]. *E. longifolia* was investigated primarily as a commercial water-extracted product (Physta^®^, Biotropics, Berhad, Kuala Lumpur, Malaysia) in seven of the nine studies included [26,27,29,30,31,32,33], with variable dosages from 100 to 600 mg/daily for a minimum of 3 days to a maximum of 6 months (Table 1).

Most of the studies (*n* = 7) reported a significant improvement in total testosterone levels after *E. longifolia* treatment [26,27,28,29,30,33,34]. However, two studies failed to observe any improvement in testosterone levels when the treatment was stopped after 3 weeks [30] or prolonged to 8 weeks [34]. In addition, Ismail et al. and George et al. did not report any difference when healthy married men were treated [31,32]. Similarly, they did not observe any difference in comparison to the placebo-controlled group [31,32]. Chinnappan et al. observed a significant difference between healthy volunteers with testosterone lower than 300 ng/dL treated with *E. longifolia* (100 mg/daily or 200 mg/daily) or placebo for 12 weeks [26]. Similar results were also described in the two PhD dissertations, including young, 18–30-year-old participants who were either sedentary males [34] or active males who trained at least 3 times/week [33]. Chan et al. reported a significant intragroup increase in testosterone with 600 mg daily treatment of *E. longifolia* over 14 days, with no significant change in the placebo group. However, no intergroup statistical analysis was provided [29]. In patients with poor Androgen Deficiency in the Aging Male (ADAM) scores, testosterone significantly increased with 200 mg daily treatment of *E. longifolia* for up to 6 months compared to placebo [28]. Furthermore, there was a significant intragroup increase in testosterone over 6 months in poor-ADAM-score patients with 200 mg daily *E. longifolia* treatment alongside concurrent exercise. However, no statistical comparison was provided to compare poor-ADAM-score patients undergoing concurrent exercise with placebo to concurrent exercise with *E. longifolia* [28].

As secondary data available in some studies, free testosterone, dehydroepiandrosterone, and SHBG results were reported. Six of the included studies analyzed free testosterone as an outcome [26,29,30,31,32,34], whereas three reported a significant intragroup increase in free testosterone with *E. longifolia* [26,29,30], with no significant improvement reported by other studies [34,35]. Interestingly, six out of nine studies reported no significant variation in SHBG levels after treatment [26,29,30,31,32,34], although Quin observed a significant improvement in SHBG levels (*p* = 0.022) when sedentary young males were supplemented with *E. longifolia* (600 mg for 8 weeks) [34]. However, no improvement was reported in comparison with placebo-controlled groups [26,29,31,32,34]. Henkel et al. and George et al. did not observe any change in dehydroepiandrosterone levels after treatment [30,32]. Conversely, Chinnappan et al. observed a slight but significant improvement in the treated group, which disappeared when compared with the placebo-controlled group [26].

Only one study reported adverse effects associated with *E. longifolia* treatment [26], which included gastrointestinal symptoms and itching, while Ismael et al. observed adverse events in both treated and placebo groups [31].

### 3.2. Study Quality of RCTs

**Figure 2:**
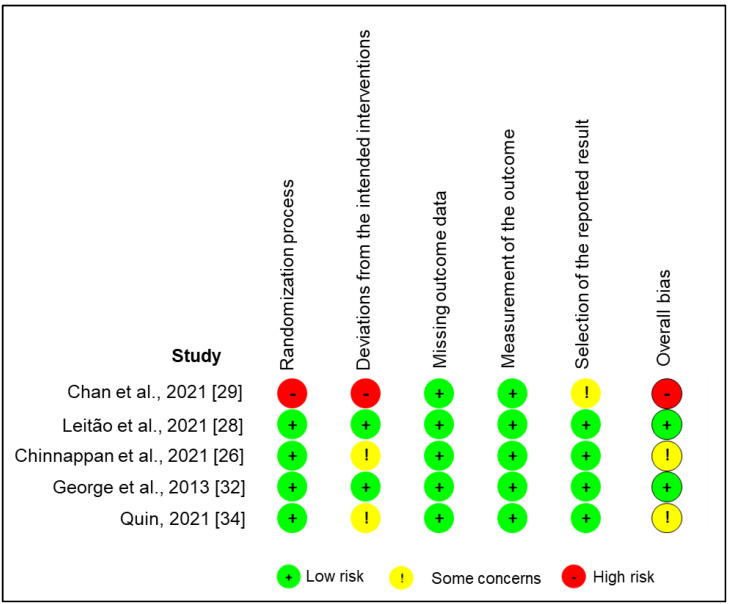
Assessment of quality and risk of bias of the studies included in the meta-analysis, using the version 2 of the Cochrane risk-of-bias tool for randomized trials [23]. Studies included: Chan et al., 2021 [29]; Leitão et al., 2021 [28]; Chinnappan et al., 2021 [26]; George et al., 2013 [32]; Quin, 2021 [34].

Five eligible RCTs measuring the testosterone levels in men (*n* = 232) were included in our meta-analysis [26,28,29,32,34]. Of these, some showed low (*n* = 2) or high (*n* = 1) risk of bias according to the Cochrane risk-of-bias tool, while others showed some concerns (*n* = 2) in the domain of deviations from the intended interventions (Figure 2).

### 3.3. Meta-Analysis

**Figure 3:**
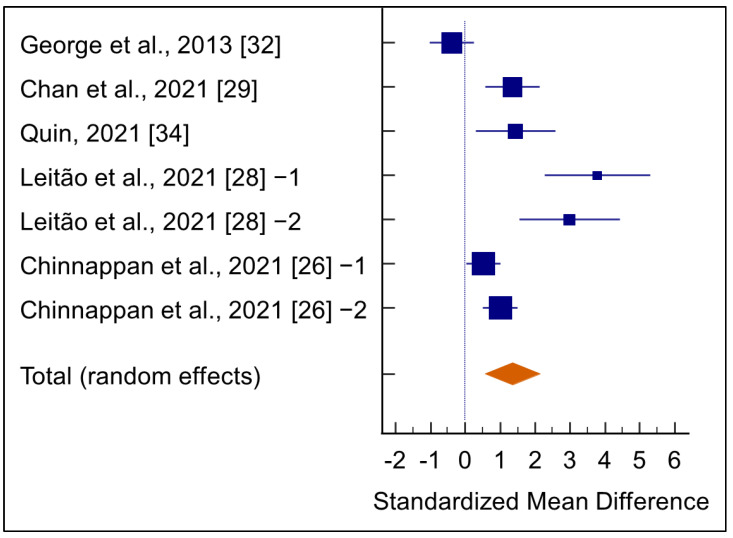
Average net change in serum testosterone levels of men supplemented with *Eurycoma longifolia*. The pooled effect size is indicated by the diamond. As different groups were analyzed in their studies after *E. longifolia* supplementation, Leitão et al., 2021 [28], and Chinnappan et al., 2021 [26], are included twice in our analysis.

Tests for heterogeneity implicated heterogeneity across the five studies (Q= 47.1472, DF= 6, *p* < 0.0001) with 87.27% inconsistency (95% CI 76.06 to 93.24). The random model effect revealed a significant increase (SMD = 1.352, 95% CI 0.565 to 2.138, *p* = 0.001) in the testosterone levels in men receiving *E. longifolia* supplementation (Figure 3 and Table 2). Furthermore, a low *p* value (0.0243) was noticed with Begg’s test, indicating publication bias (Supplementary Table S1).

**Figure 4:**
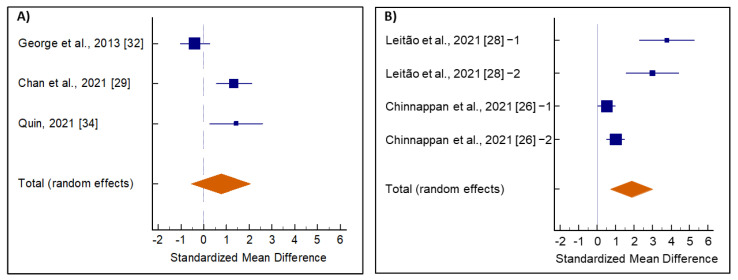
Average net change in serum testosterone levels of (**A**) normal healthy men and (**B**) men with hypogonadism supplemented with *Eurycoma longifolia*. The pooled effect size is indicated by the diamond.

Results of the subgroup analysis are presented in Figure 4 and Table 2. There were increased testosterone levels in both groups of men with (testosterone < 300 ng/dL) and without hypogonadism (testosterone > 300 ng/dL) after *E. longifolia* supplementation, although the increase was significant in the hypogonadism group. Heterogeneity was noticed among the studies in both groups (Table 2). Furthermore, Egger’s and Begg’s tests revealed significant levels of publication bias among studies included in the hypogonadism group (Supplementary Table S1).

## 4. Discussion

*E. longifolia* is reported to have anti-inflammatory and immune-regulating properties, such as improving osteoporosis, diabetes, and metabolic complications, as well as having anti-malarial, anti-anxiolytic, cytotoxic, and anti-proliferative functions in malignancy. [12,13,14]. Furthermore, *E. longifolia* may lead to increased osteoblast proliferation and apoptosis of osteoclasts. This reduces bone loss in osteoporosis, and thus, it can be considered as an alternative to TRT in these patients [36]. This systematic review highlights the beneficial use of *E. longifolia* supplementation to enhance testosterone levels, particularly in those men suffering from hypogonadism. Most of the studies included in this systematic review utilize the same commercial, freeze-dried water extract of *E. longifolia* root standardized to 0.8–1.5% eurycomanone [26,27,29,30,31,32,33]. *E. longifolia*, predominantly the roots, contains active constituents such as quassinoids, quassinoid diterpenoids, canthin-6-one alkaloids, β-carboline alkaloids, squalene derivatives, triterpene-type tirucallane, tirucallane-type triterpenes, laurycolactone, and bioactive steroids [11,12,14,15]. Derivatives of quassinoids, a group of physiologically active diterpenoids, are further classified as eurycomanones, eurycomanols, eurycomalactones, eurycolactones, eurycomanosl, and eurycomaosides [12,37]. These molecules have shown inhibitory functions in vivo and in vitro, particularly anti-inflammatory, anti-viral, anti-malarial, and anti-proliferative activities [12,15,38]. Quassinoids present in *E. longifolia* also contribute towards ergogenic effects, including increased muscle strength and endurance in cycling time, along with anxiolytic properties [37].

Eurycomanone derivatives reportedly increase testosterone levels and have anti-estrogenic activity, improving spermatogenesis [12]. Animal studies reported higher release of luteinizing hormone (LH) and follicle-stimulating hormone (FSH) gonadotropins, resulting in increased testosterone production in the Leydig cells. In addition, there may be inhibition of the aromatase enzyme, which limits its conversion to estrogen after *E. longifolia* supplementation [12,39,40]. The proprietary extraction of *E. longifolia* has also been shown to improve semen quality in infertile males over 9 months, alongside an increase in spontaneous pregnancy [41]. Similarly, a polyherbal formulation that included *E. longifolia* improved semen volume, sperm concentration, and motility after 90 days in oligozoospermic males, alongside improved serum hormone levels compared to placebo [42]. In addition to semen quality, *E. longifolia,* along with *Polygonum minus,* improved the erection hardness scale, the aging male symptom scale, and the sexual health inventory as per the diaries of men, suggesting its possible use for the treatment of male infertility [43].

Despite its impact on testosterone production, *E. longifolia* does not seem to affect the ratio between urinary testosterone glucuronide and epitestosterone glucuronide (T:E ratio), which is frequently used to determine testosterone abuse in sports doping analysis (ratio of > 6 suggests previous abuse) [44]. Therefore, since *E. longifolia* does not breech the doping policies of international sports for testosterone or precursory abuse in athletes, it is safe for consumption [45,46].

Some of the studies included in our analysis reported no or minimal side effects after *E. longifolia* treatment [26,29,30]. This is of interest because TRT, representing the first therapeutic option in the case of hypogonadism, has been associated with the presence of side effects, such as polycythaemia, fluid retention, testicular atrophy, prostate enlargement, congestive heart failure, and obstructive sleep apnea [47]. Furthermore, in males and females, *E. longifolia* does not negatively affect AST/ALT or body weight, further supporting safe treatment with *E. longifolia* [48]. Therefore, *E. longifolia* extract may represent a promisingly safe treatment option for hypogonadism.

Although this meta-analysis suggests a possible use of *E. longifolia* supplementation to improve testosterone production, important limitations are to be highlighted. First and foremost, the analysis was based on five studies, which were heterogeneous in terms of study design, included population, dosage and length of treatment, and limited sample sizes. However, the inclusion of randomized controlled trials using *E. longifolia* as a sole intervention, as well as the Cochrane standardized tool for assessing quality of evidence, may represent the strength of this analysis since conclusions are based on quality publications, with just one study showing high risk of bias. In addition, most of the studies discussed in this systematic analysis used the same commercial product for *E. longifolia* supplementation, therefore limiting the potential influence of other components on the investigated outcomes. Importantly, the quality of *E. longifolia* can be determined based on eurycomanone concentrations, with a recommended level of 0.8–1.5 *w/v* (%) [49]. However, not all products on the market meet these requirements. Out of 41 products containing *E. longifolia* as a single or compound formulation from Malaysia, 24 products contained eurycomanone: 11/24 reached the recommended levels, while 9 were above the recommended levels (1.6–8.48% *w/v*). Some products did not contain any eurycomanone [49].

## 5. Conclusions

In conclusion, this systematic analysis of the literature highlights the possible use of *E. longifolia* supplementation for enhancing testosterone production. Although more research is required before its use in clinical practice, this may represent a safe and promising therapeutic option, particularly for patients with hypogonadism.

## Figures and Tables

**Table 1:** Characteristics of the studies investigating the effect of *E. longifolia* on serum testosterone levels in men.

Reference	Study Design	Study Population	EL Supplement	Dosage	Testosterone (ng/dL) [Mean ± SD]		*p* Value *
					Treatment Group	Placebo Group	
Chan et al., 2021 [29]	Double-blind, controlled trial	Healthy (18–30 y) men with no diagnosis of hypogonadism (*n* = 16) vs. controls (*n* = 16)	Physta, Biotropics	600 mg/day for 2 weeks	Pre: 802 ± 160 Post: 924 ± 84	Pre: 791 ± 150 Post: 769 ± 135	N/A
Leitão et al., 2021 [28]	Double-blind RCT	Patients with ADAM, 40–59 y (*n* = 9) vs. controls (*n* = 12)	Dry extract	200 mg up to 6 months	Pre: 278.2 ± 20.5 Post: 400.3 ± 38.9	Pre: 281.5 ± 17.7 Post: 258.5 ± 33.7	*p* < 0.05
		Patients with ADAM, 40–59 y, with concurrent exercise (*n* = 9) vs. controls (*n* = 7)			Pre: 253 ± 20.5 Post: 374.5 ± 38.9	Pre: 286.7 ± 21.7 Post: 370.8 ± 41.3	N/A
Chinnappan et al., 2021 [26]	Double-blind, multicenter RCT	Healthy volunteers: 50–70 y (*n* = 35) vs. controls (*n* = 35)	Physta, Biotropics	100 mg/daily up to 12 weeks	Pre: 187.3 ± 46.4 Post: 203.8 ± 54.6	Pre: 183.0 ± 37.8 Post: 177.9 ± 43.7	*p* < 0.05
		Healthy volunteers: 50–70 y (*n* = 35) vs. controls (*n* = 35)		200 mg/daily up to 12 weeks	Pre: 200.5 ± 46.4 Post: 225.0 ± 49.8	Pre: 183.0 ± 37.8 Post: 177.9 ± 43.7	*p* < 0.05
Quin, 2021 [34]	Double-blind RCT	Sedentary males (18–30 y) (*n* = 8) vs. controls (*n* = 8)	N/A	600 mg for 2 weeks	Pre: 871 ± 200 Post: 968 ± 70	Pre: 863 ± 150 Post: 790 ± 150	*p* < 0.05
		Sedentary males (18–30 y) (*n* = 11) vs. controls (*n* = 10)		600 mg for 8 weeks	Pre: 685 ± 240 Post: not reported	Pre: 725 ± 170 Post: not reported	N/S
Lim, 2017 [33]	Double-blind RCT	Men trained at least 3 times/week, 18–30 y, BMI: 18.5–25.0 (*n* = 9) vs. controls (*n* = 11)	Physta, Biotropics	1.7 mg/kg of body weight for 3 days	Pre: 0.63 mmol/L Post: 0.86 mmol/L	Pre: 0.82 mmol/L Post: 0.59 mmol/L	*p* < 0.05
				1.7 mg/kg of body weight for 5 weeks	Pre: 0.63 mmol/L Post: 1.26 mmol/L	Pre: 0.67 mmol/L Post: 0.83 mmol/L	*p* < 0.05
Henkel et al., 2014 [30]	Pre- vs. Post-	Male cyclists (57–72 y), with or without chronic diseases associated with age (*n* = 13)	Physta, Biotropics	400 mg for 3 weeks	Pre: 384 ± 79 Post: 409 ± 102	N/A	N/A
				400 mg for 5 weeks	Pre: 384 ± 79 Post: 442 ± 115	N/A	N/A
Tambi et al., 2012 [27]	Pre- vs. Post-	Patients with hypogonadism and LOH (*n* = 76)	Physta, Biotropics	200 mg up to 1 month	Pre: 163 ± 43.5 Post: 240 ± 71.2	N/A	N/A
Ismail et al., 2012 [31]	Double-blind RCT	Healthy married men, 30–55 y, with or without stable chronic medical illnesses (*n* = 54) vs. controls (*n* = 55)	Physta, Biotropics	300 mg for 12 weeks	Pre: 476 ± 167 Post: 435 to 479	Pre: 542 ± 133 Post: 522 to 549	N/S
George et al., 2013 [32]	Double-blind RCT	Healthy men (*n* = 21) vs. controls (*n* = 19)	Physta, Biotropics	300 mg for 12 weeks	Pre: 458 ± 152.1 Post: 484 ± 165.3	Pre: 540 ± 177 Post: 542 ± 187	N/S

N/A: not available; N/S: not significant. *: *p* value is reported for treatment vs. placebo group.

**Table 2:** Meta-analysis of the outcome variable testosterone in serum.

Study	EL Group (*n*)	CTRL (*n*)	Tot (*n*)	SMD	SE	95% CI	t	*p*	Random Weight (%)
**Studies evaluating testosterone levels in men supplemented with *Eurycoma longifolia***									
George et al., 2013 [32]	21	19	40	−0.388	0.313	−1.022 to 0.247			15.84
Chan et al., 2021 [29]	16	16	32	1.344	0.383	0.561 to 2.127			15.10
Quin et al., 2021 [34]	8	8	16	1.438	0.537	0.287 to 2.589			13.33
Leitão et al., 2021-1 * [28]	9	12	21	3.783	0.721	2.274 to 5.292			11.16
Leitão et al., 2021-2 * [28]	11	7	18	2.983	0.678	1.546 to 4.419			11.66
Chinnappan et al., 2021-1 * [26]	35	35	70	0.518	0.240	0.0382 to 0.998			16.50
Chinnappan et al., 2021-2 * [26]	35	35	70	0.994	0.251	0.494 to 1.495			16.41
**Total (random effects)**	135	132	267	1.352	0.399	0.565 to 2.138	3.384	0.001	100.00
**Q = 47.1472, DF = 6, *p* < 0.0001, I^2^ = 87.27% (95% CI 76.06 to 93.24)**									
**Studies evaluating testosterone levels in normal healthy men supplemented with *Eurycoma longifolia***									
George et al., 2013 [32]	21	19	40	−0.388	0.313	−1.022 to 0.247			35.39
Chan et al., 2021 [29]	16	16	32	1.344	0.383	0.561 to 2.127			34.02
Quin et al., 2021 [34]	8	8	16	1.438	0.537	0.287 to 2.589			30.59
**Total (random effects)**	45	43	88	0.760	0.654	−0.540 to 2.060	1.162	0.249	100.00
**Q = 15.9216, DF = 2, *p* = 0.0003, I^2^ = 87.44% (95% CI 64.47 to 95.56)**									
**Studies evaluating testosterone levels in men with hypogonadism supplemented with *Eurycoma longifolia***									
Leitão et al., 2021-1 * [28]	9	12	21	3.783	0.721	2.274 to 5.292			5.16
Leitão et al., 2021-2 * [28]	11	7	18	2.983	0.678	1.546 to 4.419			5.84
Chinnappan et al., 2021-1 * [26]	35	35	70	0.518	0.240	0.0382 to 0.998			46.40
Chinnappan et al., 2021-2 * [26]	35	35	70	0.994	0.251	0.494 to 1.495			42.60
**Total (random effects)**	90	89	179	1.861	0.579	0.719 to 3.002	3.217	0.002	100.00
**Q = 27.4384, DF = 3, *p* < 0.0001, I^2^ = 89.07% (95% CI 74.70 to 95.27)**									

SMD: standard mean difference; SE: standard error; CI: confidence interval. * As different groups were analyzed in their studies after *E. longifolia* supplementation, Leitão et al., 2021 [28], and Chinnappan et al., 2021 [26], are included twice in our analysis.

## Data Availability

The scientific studies analyzed in this meta-analysis are available in the databases PubMed, Scopus, Web of Science, Cochrane, Ovid/Embase, and Google Scholar.

## Supplementary Materials

The following supporting information can be downloaded at: https://www.mdpi.com/1648-9144/58/8/1047/s1; Table S1: Publication bias across studies included in meta-analysis.
