# Supplementary material for: Eurycoma longifolia (Jack) Improves Serum Total Testosterone in Men: A Systematic Review and Meta-Analysis of Clinical Trials
Source: Medicina (Kaunas). 2022 Aug 4;58(8):1047. doi: 10.3390/medicina58081047 (PMC9415500; doi:10.3390/medicina58081047)
Supplement: Supplementary file 1 [file medicina-58-01047-s001.zip › medicina-1815143-supplementary.pdf]

Supplementary Table S1: Publication bias across studies included in meta-analysis

Study Types	Egger's test			Begg's test	
	Intercept	95% CI	P value	Kendall's Tau	P value
**Studies (n=5) evaluating testosterone levels in men supplemented with *E. longifolia***	5.1508	-0.2908 to 10.5925	0.0591	0.7143	0.0243
**Studies (n=3) evaluating testosterone levels in normal healthy men supplemented with *E. longifolia***	8.7118	-78.0781 to 95.5016	0.4233	0.3333	0.6015
**Studies (n=2) evaluating testosterone levels in hypogonadism men supplemented with *E. longifolia***	5.8527	1.2257 to 10.4796	0.0321	1.0000	0.0415
